# Adult Stem Cell Therapeutics in Diabetic Retinopathy

**DOI:** 10.3390/ijms20194876

**Published:** 2019-09-30

**Authors:** Sriprachodaya Gaddam, Ramesh Periasamy, Rajashekhar Gangaraju

**Affiliations:** 1 Department of Ophthalmology, University of Tennessee Health Science Center, College of Medicine, Memphis, TN 38163, USA; sg3538@drexel.edu (S.G.); lettersforramesh@gmail.com (R.P.); 2 Department of Anatomy & Neurobiology, University of Tennessee Health Science Center, College of Medicine, Memphis, TN 38163, USA

**Keywords:** inflammation, hypoxia, diabetes, retina, adipose stem cells, mesenchymal, paracrine, CD34, CD140b

## Abstract

Diabetic retinopathy (DR), a complication of diabetes, is one of the leading causes of blindness in working-age adults. The pathology of the disease prevents the endogenous stem cells from participating in the natural repair of the diseased retina. Current treatments, specifically stem cell therapeutics, have shown variable efficacy in preclinical models due to the multi-faceted nature of the disease. Among the various adult stem cells, mesenchymal stem cells, especially those derived from adipose tissue and bone marrow, have been explored as a possible treatment for DR. This review summarizes the current literature around the various adult stem cell treatments for the disease and outlines the benefits and limitations of the therapeutics that are being explored in the field. The paracrine nature of adipose stem cells, in particular, has been highlighted as a potential solution to the lack of a homing and conducive environment that poses a challenge to the implantation of exogenous stem cells in the target tissue. Various methods of mesenchymal stem cell priming to adapt to a hostile retinal microenvironment have been discussed. Current clinical trials and potential safety concerns have been examined, and the future directions of stem cell therapeutics in DR have also been contemplated.

## 1. Diabetes and Diabetic Retinopathy

In diabetes, the body does not properly process glucose for use as energy resulting in serious health complications associated with heart disease, kidney failure, and blindness [1]. Diabetes was reported as the seventh leading cause of death in the United States in 2015, with the rate of diagnoses increasing with age [2]. Diabetic retinopathy (DR), a diabetes complication that causes damage to the blood vessels in the light-sensitive tissue of the retina. Consequently, it is a leading cause of blindness in all working-age adults and the most common cause of vision loss among people with diabetes [3]. The hyperglycemia that is characteristic of poorly controlled diabetes triggers the mechanisms that cause DR which encompasses both genetic and epigenetic factors, as well as increased production of free radicals, inflammatory factors, advanced glycosylation end products, and vascular endothelial growth factor (VEGF) [4]. DR is classified into two stages, non-proliferative DR (NPDR) and proliferative DR (PDR). Early stages of the disease are characterized by inflammation, microglial activation, loss of endothelial cells, neuronal cells, and pericytes, and damage to the integrity of the retinal microvasculature [5]. As the disease progresses, macular edema can develop and lead to subsequent vision loss, and local ischemia can lead to vision-threatening PDR [5]. While treatments are available for later stages of DR, the only therapeutic strategy clinicians can currently offer patients for the early stages is the strict control of blood glucose and blood pressure as a way to manage risk factors for the disease [6]. Treatment options for advanced DR are laser photocoagulation, intraocular anti-VEGF, or glucocorticoid drugs, all of which can minimize vision loss if conducted promptly [5]. Currently, three FDA-approved anti-VEGF agents are being used to treat DR and are delivered via an injection into the vitreous gel to reverse abnormal blood vessel growth and decrease fluid accumulation within the retina [3]. While the discovery of VEGF inhibitors revolutionized the management of DR, not all patients respond to this treatment due to the multifaceted and complex nature of the condition [7]. In fact, anti-VEGF therapy in DR is associated with severe complications including endophthalmitis, intraocular inflammation, rhegmatogenous retinal detachment, tractional retinal detachment, intraocular pressure elevation, ocular hemorrhage, and ghost cell glaucoma [8]. Glucocorticoid use in the eye increases the risk of cataract and glaucoma and therefore patients must be monitored for increased eye pressure and signs of glaucoma [3]. Therefore, due to the adverse side effects and variable efficacy of current treatments, research labs across the US are seeking better ways to treat DR with new therapies, including stem cell therapy, which are being tested and compared to known treatments.

## 2. Adult Stem Cells

**Figure 1:**
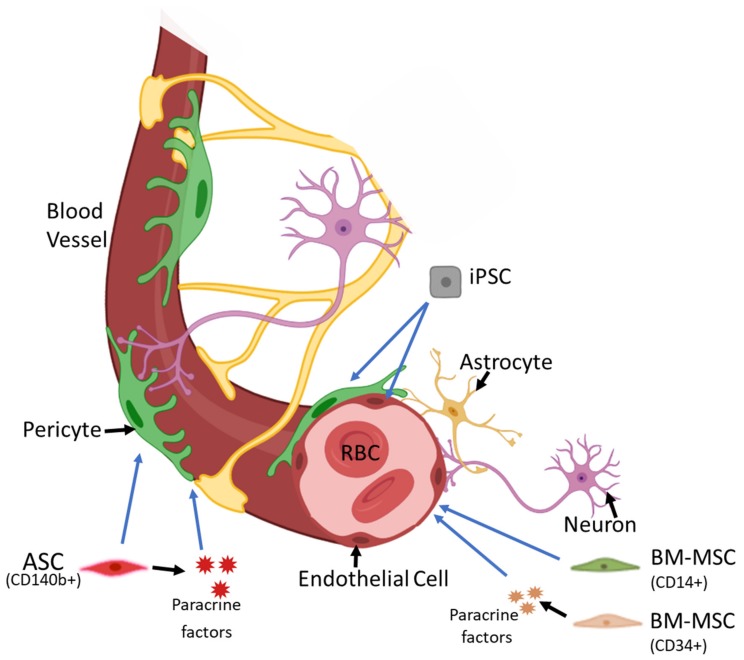
Current regenerative cell therapy efforts in diabetic retinopathy. A variety of stem cells including bone marrow mesenchymal stem cells (BM-MSC), adipose stem cells (ASC), and induced pluripotent stem cells (iPSC) have been considered in diabetic retinopathy. Arrows point to the stem cells that either differentiates into endothelial or pericyte like cells to replace lost cells or provide trophic support (Please refer to the text for more details). The image was adapted with permission from Springer Nature: Regenerative Medicine—from Protocol to Patient. Regenerative Therapies for Retinopathy. Periasamy R., Gangaraju R. (2016), designed using Adobe Photoshop and drawn in Adobe Illustrator.

Several stem cell therapies are being intensely researched as possible treatments for degenerative eye diseases, including DR (Figure 1). Due to the involvement of cell loss in the progression of the disease, cell replacement via stem cell therapy has been a topic of interest among researchers. Embryonic or induced pluripotent stem cells (iPSC), hematopoietic stem cells, endothelial progenitor cells, and mesenchymal stromal cells (MSC) have all been used in preclinical models for the treatment of DR [5].

Adipose stem cells (ASC) and bone marrow-derived mesenchymal stem cells (BM-MSC) are types of MSCs that have been shown to bear promise in the regeneration and recovery of the retina once it has been damaged. MSCs from these tissues have been shown to have a neuroprotective effect in the rat experimental ocular hypertension (OHT) model and have been promising in the treatment of glaucoma [9]. MSCs have also been reported to potentially be a useful source of paracrine factors that can protect retinal ganglion cells (RGCs) and aid in the regeneration of the optic nerve in some degenerative eye diseases [10].

ASCs have been demonstrated in vivo to be functionally and phenotypically similar to the pericytes lining microvessels in adipose tissues and also promote angiogenesis, improve ischemia, and offer protection against nerve damage via paracrine factors or through physical contact with endothelial cells [11]. ASCs have shown the potential to be therapeutically effective in early-stage DR in preclinical rat models as injection of ASCs to the rat eyes showed improved retinal function, measured by electroretinogram, and a significant decrease in vascular leakage and apoptotic cells around the retinal vessels, shown through retinal histopathological evaluation [12]. ASCs have also demonstrated an ability in mouse models of DR to differentiate into pericytes, posing the possibility of retinal vasculature repair [13]. Intravitreal administration of ASCs can also trigger an effective cytoprotective microenvironment in the retina of diabetic mice by reducing oxidative damage and increasing the intraocular levels of several potent neurotrophic factors including nerve growth factor, basic fibroblast growth factor and glial cell line-derived neurotrophic factor [14]. In addition, the ASCs administration completely prevented RGC loss which is an early event in the onset of DR, offering hope to the treatment of the early stages of the disease [14]. ASCs have also been shown to improve the BRB integrity in diabetic rats by differentiation into photoreceptor and glial-like cells in the retina, suggesting a promising therapeutic approach to repair the damaged neurovascular unit [15]. The differentiation of ASCs depends on numerous factors that are yet to be fully investigated although it appears that ASC differentiation is affected by the age of the donor, culture methods, isolation procedures, and addition of specific cocktails of chemical inducers and/or cytokines [16].

Hematopoietic stem cells (HSC) and progenitor cells (PC) are also involved in retinal microvascular repair during diabetes, making them an essential part of the bone marrow microenvironment [17]. One upcoming approach in the treatment of DR is to pharmacologically protect the bone marrow HSC/PC populations from the oxidative stress and advanced glycation end-product accumulation that occurs as a result of diabetes [17]. A recent study further demonstrated the importance of HSC/PC preservation in DR by showing that the loss of angiotensin-converting enzyme 2, the primary enzyme in of the vasoprotective axis of the renin-angiotensin system (RAS), promotes bone marrow dysfunction by reducing the number of repopulating hematopoietic stem cells, which in turn increased the progression of DR [18]. Therefore, a possible therapeutic avenue for DR may be to support the activation of protective RAS within HSC/PCs to maintain bone marrow quality.

BM-MSCs are being explored as a therapeutic option for retinal regeneration due to the apparent plastic nature of hematopoietic stem cells from the bone marrow and their role in repopulating bone marrow in transplant recipients and repairing vasculature [19]. Endothelial progenitor cells positive for CD34, some of which differentiate into endothelial cells, function primarily to repair vasculature through paracrine mechanisms be well tolerated and were efficient in vascular repair in animal models [20,21,22]. However, it has been shown that the bone marrow of diabetic patients may contain a high concentration of CD34+ endothelial progenitor cells that are trapped in the bone marrow due to the diabetic pro-inflammatory environment, making the use of these cells to treat DR a challenge [23]. In another study, a subgroup of bone marrow-derived circulating angiogenic cells, CD14+ cells, have demonstrated the ability to affect the vascular repair and could potentially be an avenue for DR cell therapy [24]. Interestingly, intravitreally injected BM-MSCs were found to integrate into the inner retina and differentiate into retinal glial cells and improve ERG amplitude thereby protecting vision [25], although another study did not find any benefit on ERG [22]. Despite these promising results, the efficacy and potential mechanism of BM-MSC therapy are questionable since a long-term safety study revealed that some of the human bone marrow cells integrated into other ocular structures and circumvented the blood-retinal barrier to migrate into non-target tissue in a similar DR rat model [26].

MSCs from less well-studied tissues such as derived from dental pulp also possess many of the in vitro features of BM-MSCs including clonogenicity, expression of certain markers, and eventual differentiation into osteoblasts, chondrocytes and adipocytes after stimulation, which hold therapeutic potential in promoting neuroprotection and axon regeneration of RGCs after optic nerve injury although not described in DR models [27]. On the other hand, umbilical cord-derived mesenchymal stem cells (UC-MSCs) have been implicated as a possible therapeutic option in the treatment of the neurodegeneration that occurs in DR by increasing the number of surviving RGCs [28]. In one study, treatment of diabetic rats with neural stem cells (NSCs) derived from UC-MSCs showed morphological improvements, which translated into a restoration of vision documented by a focal ERG [28]. In addition. Wharton’s jelly, a gelatinous substance found in the umbilical cord, contains UC-MSCs that reduces axotomy-induced RGC loss through the secretion of neuroprotective and anti-inflammatory factors [29]. Human cord blood-derived endothelial colony-forming cells (ECFCs) are another type of adult stem cells that showed promise in treating ischemic retinopathies and have undergone preclinical evaluation and optimization [30]. Finally, human placental amniotic membrane-derived MSCs (AMSCs) also hold promise in the treatment of DR due to the retinal angiogenic effects of the TGF-β1 and paracrine factors that are secreted from the cells in response to the pathological environment of DR [31].

## 3. Stem Cell Alterations

While endogenous retinal stem cells may have the potential to replace damaged or lost RPE cells and photoreceptors, treatment of injured RGCs has been managed with the use of MSCs due to their ability to generate paracrine factors [10]. Based on current evidence, endogenous stem cells do not provide significant paracrine support for the stimulation of retinal regeneration as their mechanism of action is typically restricted to RPE cell and photoreceptor replacement, highlighting the need for exogenous stem cells to fill the gaps in retinal disease therapy [10]. The main appeal of MSCs is their potential allogeneic use as these cells do not express HLA class antigens II and low levels of HLA class I antigens [32]. MSCs have been shown in numerous studies to have an immunosuppressive role as they fail to stimulate allogeneic lymphocyte proliferation, remain immunosuppressive despite IFN-γ stimulation, generate regulatory T-cells, and secrete cytokines [32]. Considering the role that inflammation plays in the progression of DR, MSCs have gained notable interest in their potential to regenerate tissue through their immunosuppressive qualities.

**Figure 2:**
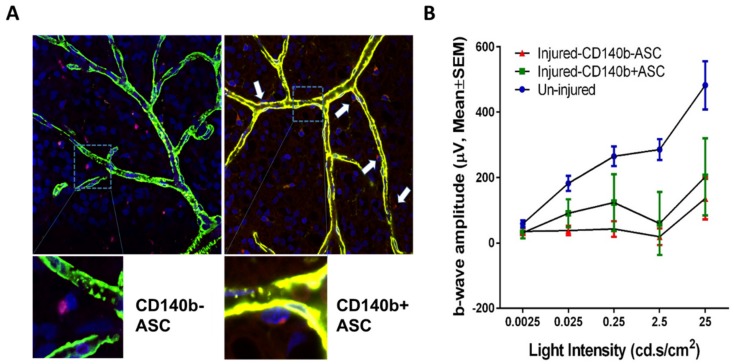
Intravitreal injection of CD140b+ ASC localize on the host retinal vasculature and improved visual function better than the CD140b- ASC. Unilateral retinal I/R was done in adult Lewis rats by transiently elevating the IOP for 1 h. On day 7 of reperfusion, the animals were randomized to receive intravitreal CD140b+ASC, CD140b-ASC (1000 cells/eye) or saline injections. (**A**) Confocal image of the retinal flat mount of retinal I/R injury rat with CD140b-ASC (left) and CD140b+ASC (right) after 21 days. Arrows point to maurocalcine labeled ASC (red). Blood vessels were identified using Griffonia Simplicifolia Lectin I (GSL I) isolectin B4 (green) and counterstained with DAPI-labeled nucleated cells (blue). 20× magnification. Inset box is the enlarged portion showing CD140b+ASC on the vasculature. (**B**) Retinal I/R resulted in a reduction in “b” wave amplitude, which was improved by CD140b+ ASC compared with CD140b- ASC (25cd.ms^2^, *p* < 0.0001). Data shown is a representation of 3–7 animals/group.

However, a significant challenge in the use of MSC therapy is the lack of specific homing of exogenous stem cells and the limitation of current knowledge on the mechanisms of homing of various types of MSCs. One important method that is being studied and refined is the pretreatment or priming of MSCs in culture to increase CXCR4, a homing molecule expression on the membrane through the addition of cytokines or cytokine cocktails to the culture medium [33]. These cytokines can include IFN-γ, TNF- α, both of which enhance the immunosuppressive properties of MSCs, and IL-17, a proinflammatory cytokine [34]. It is currently known that ASCs migrate through tissue mainly through Stromal derived factor-1 (SDF-1)/CXCR4 and CXC ligand-5 (CXCL5)/CXCR2 interactions [35]. Several other signaling pathways are also involved including LPA/LPA1 signaling pathway, MAPK/Erk1/2 signaling pathway, RhoA/Rock signaling pathway and PDGF-BB/PDGFR-β signaling pathway [35]. Since ASCs share phenotypic overlap with pericytes lining microvessels, we have recently shown knockdown of PDGFR-β (CD140b) signaling in ASCs impairs the vascular network formation with retinal endothelial cells in vitro, suggesting a pivotal role for CD140b in the intrinsic abilities of ASCs and their angiogenic influence on retinal endothelium [36]. Although the cell surface expression of CD140b is not a bona fide ASC marker [37], regardless of passage number it is constitutively expressed in most ASC [38,39] and dynamically ranged from 40–70% in cell culture [40]. Taking advantage of this, we have recently isolated CD140b positive ASCs using the FACS sorting method and assessed their therapeutic ability in a rat retinal ischemia-reperfusion injury (I/R) model. Interestingly, intravitreal injection of CD140b+ASC was better at homing to the retina as well as improved the b-wave amplitude, a measure of inner retinal response suggesting a therapeutic benefit of these specific population of stem cells (Figure 2).

Diabetes causes a decrease in the phosphorylation and intracellular redistribution of vasodilator-stimulated phosphoprotein (VASP), an actin motor protein needed for cell migration, which in turn causes the homing of CD34+ cells from the peripheral blood of diabetic human patients to be defective [41]. This is a significant hurdle in the use of these cells to treat DR since diabetic patients cannot be treated with their own CD34+ cells due to their defective nature and yet using donor CD34+ cells would pose a severe risk of immune-rejection. A possible strategy to overcome this defect is to co-administer CD34+ cells with MSCs since they have been shown to enhance the migration and colocalization of CD34+ cells with retinal vessels due to their immune modulation abilities [24]. On the other hand, CD14+ cells appear to retain the homing characteristic in diabetic subjects due to mechanisms that are yet to be fully understood [24]. Lack of proper homing to the tissue that needs to be repaired will render the transplanted stem cells incapable of repairing vascular damage and subsequently unable to prevent further disease progression. Therefore, further studies and subsequent development of strategies to improve the homing of stem cells are needed for increased clinical efficiency. Future studies need to consider ways to re-engineer the cells to express some of the above proteins to improve upon the homing ability of stem cells in vivo. Towards this end, one option we are considering is to introduce or knockdown specific proteins in ASCs via the use of clustered regularly interspaced short palindromic repeats (CRISPR)-based epigenome editing which has been successfully shown for ASCs [42]. Additionally, the use of cord blood-derived iPSC differentiated into vascular progenitors expressing endothelial CD31 (cluster of differentiation 31 or platelet endothelial cell adhesion molecule) and markers found on pericytes such as CD146 (cluster of differentiation 146 or melanoma cell adhesion molecule), may be useful in the treatment of DR since these cells intravitreally injected into ischemia-reperfusion injury model demonstrated pericyte localization while intravenously injected were found to be localized primarily in endothelial positions [43].

## 4. Paracrine Nature of Stem Cells

The mechanism by which MSCs regenerate and repair retinal tissue appears to be through the secretion of paracrine factors, offering a means to bypass the issues of using live stem cells—cell viability in vivo, lack of homing and integration into target retinal tissue, and inability to properly function in a potentially pro-inflammatory environment [44]. ASCs specifically is an attractive option for paracrine mediated therapy due to their high abundance in tissue and the ability to differentiate into pericytes, which are essential in the retinal vasculature. ASCs have been shown to produce a variety of growth factors, cytokines, and chemokines, providing trophic immunosuppressive and anti-inflammatory effects. They also show the capability to withstand bioenergetic changes induced by hyperglycemia [45]. In hyperglycemic conditions, despite the increased level of apoptosis, the proliferation rate of ACSs was not affected, and in vitro, they maintained their ability to promote the formation of vascular-like networks of human umbilical vein endothelial cells [45].

Prolonged exposure of endogenous MSCs to a pathological microenvironment in vivo reduces their ability to respond to environmental cues. Therefore, the priming of these cells with various cytokines and other biomolecules poses a potential solution to improving paracrine function in a toxic microenvironment [34]. Recently our group showed that ASCs or their secreted factors were able to diminish retinal complications of diabetes in the Ins2^Akita^ mouse model of DR [46]. In this study, the visual acuity of mice injected with condition media from cytokine-primed ASCs was found to be significantly improved similar to that of mice who received an intravitreal injection of CD140b-positive ASCs [46]. More importantly, cytokine-primed ASC conditioned medium demonstrated protection against vascular leakage in vivo as well as against the TNF-α induced endothelial permeability in vitro suggesting that ASC produced paracrine factors affect the integrity of vascular endothelial cells [46]. Among the many proteins produced, anti-inflammatory proteins such as IDO-1, IDO-2, and TSG-6 were shown to be highly expressed in the cytokine-primed ASC condition media, demonstrating a potential therapeutic benefit to the priming process of ASCs [46].

MSCs can also be primed with antioxidants to increase the effectiveness of their paracrine nature as shown in a study where the effect of antioxidant preconditioning on both the molecular mRNA and protein levels were analyzed [47]. The data from the study suggested that the combined ex vivo treatment of autologous stem cells with N-acetylcysteine and ascorbic acid 2-phosphate could potentially be an effective method of restoring the paracrine function of impaired diabetic MSCs [47]. Pre-conditioning ASCs with LL-37, a naturally occurring antimicrobial peptide involved in wound repair, has been shown to increase early growth response 1 (EGR1) expression and MAPK activation which can aid in ASC cell expansion, migration, and paracrine nature [48]. Hypoxia-primed murine MSCs have also shown upregulation of pro-survival genes, including enhanced survival of engrafted cells, and increased secretion of anti-oxidant, anti-apoptotic, and growth factors, all of which can aid in MSC engraftment, survival in the ischemic environment, and angiogenic potential [44].

## 5. Clinical Trials

Currently, multiple clinical trials are aiming to test stem cell-mediated therapy for DR and other ocular diseases. Human clinical trials are being conducted with retinal stem cell transplantation using pluripotential stem cells (PSC) and iPSCs that share morphological similarities with photoreceptors and retinal pigment epithelial (RPE) cells [44]. Some trials involve the use of adipose or bone marrow-derived stem cells were directly transplanted into the eye to mend retinal damage in patients with age-related macular degeneration (AMD) and other blinding disorders [44]. In one ongoing study (NCT01736059), intravitreal injections of CD34+ Bone marrow MSCs are being administered to subjects with irreversible vision loss from retinal degenerative diseases or retinal vascular disease, including DR. Another clinical trial (NCT03403283) is underway to determine whether or not endothelial progenitor cells (EPC’s) are defective in people with diabetes to further understand the mechanisms of the disease. A third clinical trial (NCT03403699) is examining the abilities of iPSCs to generate endothelial cells and pericytes in areas with capillary degeneration seen in DR.

The growing popularity of stem-cell clinics in the United States highlights some major concerns in the use of ASCs to treat degenerative eye diseases as three patients who received the treatment for AMD developed severe bilateral vision loss as a result of the associated complications of ocular hypertension, hemorrhagic retinopathy, vitreous hemorrhage, combined traction, and rhegmatogenous retinal detachment, or lens dislocation [49]. However, the issue with the stem cell therapy in these cases appears to be due to the lack of homogeneity in the sources used to harvest these cells and the methods used to administer them to the patients [50]. Therefore, while patients and physicians must exercise caution when considering treatments at commercial cell therapy clinics, the merits of stem cell therapy itself should not be wholly discredited. To support this notion, recently autologous bone marrow-derived MSCs were found to be beneficial in NPDR subjects with significant improvements in macular thickness and improvement in best-corrected visual acuity (BCVA) from baseline [51].

## 6. Limitations of Stem Cell Therapies and Future Directions

Although we have made tremendous progress in understanding stem cell therapies in DR and other diabetic complications, there are several challenges to a clinical translation that need to be addressed soon in the near future. One of the major obstacles of MSC treatment is that the hostile environment of diseased retina is often not conducive to homing due to the toxic pathology of DR. Currently, the steps being taken to improve the homing efficiency of MSCs include developing modifications to mode of administration, optimizing culture conditions for greater expression of homing molecules, and engineering of cell surface receptor or the target tissue itself to improve homing [33]. While some cells specifically ASCs have been shown to be resistant to hyperglycemia [12,45,52], others have demonstrated diabetic ASCs are functionally impaired compared with healthy ASCs [53] suggesting conflicting data that requires additional studies to better understand how exogenously injected stem cells for repair could be manipulated for better outcomes. Another hurdle that must be crossed in stem cell therapy is problems with limited proliferation and differentiation ability for certain cell types such as retinal progenitor cells [54]. Towards this end, the use of decellularized ASC matrix [54] or de-differentiation of RPE via the use of retroviral transduction in reprogramming to generate terminally differentiated cells of the neural retina has shown promise [55]. Another translational challenge of preclinical studies of stem cell therapeutics is the difference between animal models and human disease with clinical endpoints unmatched while also the clinical parameters not predictive of outcomes of such therapies [56]. Specifically, proper correlations of histopathology, molecular abnormalities with suitable and clinically validated endpoints relevant to humans are necessary. Toward this end, the use of integral hematologic parameters has been shown to predict the outcomes after autologous MSC transplantation [57] though these parameters may not represent tissue or organ-specific function. It is unclear if a single stem cell type or combination of various cell types is necessary to develop effective therapies. To explore this avenue, there is great interest in the development of ex vivo expanded adult stem cell-derived retinal organoids [58]. The use of such organoids is expected to retain their tissue identity and genome stability for an effective therapeutic outcome. The organization of such 3D retinal structures using iPSC is well documented although the denervation and microcirculation are still an open challenge. Last but not least, translation of laboratory stem cell therapeutics must be scaled for human use, and thus the challenges of preparing cells under GMP conditions and the reproducibility of manufacturing conditions to meet the specifics of regulatory authorities need to be addressed. Despite the odds, regenerative medicine has seen remarkable progress in the last few years for stem cell therapeutics in retinal disorders, and we expect this trend will continue over the next few years to result in a successful outcome.
